# Non-Invasive and Quantitative Evaluation for Disuse Muscle Atrophy Caused by Immobilization After Limb Fracture Based on Surface Electromyography Analysis

**DOI:** 10.3390/diagnostics14232695

**Published:** 2024-11-29

**Authors:** Lvgang Shi, Yuyin Hong, Shun Zhang, Hao Jin, Shengming Wang, Gang Feng

**Affiliations:** 1 Polytechnic Institute, Zhejiang University, Hangzhou 310015, China; slgslg@zju.edu.cn (L.S.); hongyuyin@zju.edu.cn (Y.H.); 2 College of Information Science & Electronic Engineering, Zhejiang University, Hangzhou 310027, China; shunzhang@zju.edu.cn (S.Z.); hjin@zju.edu.cn (H.J.); 3 International Campus, Zhejiang University, Haining 314400, China; 4 2nd Affiliated Hospital, School of Medicine, Zhejiang University, Hangzhou 310009, China

**Keywords:** disuse muscle atrophy, surface electromyography, frequency band of power spectrum density, composition of muscle fiber types

## Abstract

Background: The clinical evaluation for disuse muscle atrophy usually depends on qualitative rating indicators with subjective judgments of doctors and some invasive measurement methods such as needle electromyography. Surface electromyography, as a non-invasive method, has been widely used in the detection of muscular and neurological diseases in recent years. In this paper, we explore how to evaluate disuse muscle atrophy based on surface electromyography; Methods: Firstly, we conducted rat experiments using hind-limb suspension to create a model of disuse muscle atrophy. Five groups of rats were suspended for 0, 3, 7, 14, and 21 days, respectively. We induced leg electromyography of rats through electrical stimulation and used fluorescence staining to obtain the fiber-type composition of rats’ leg muscles. We obtained the best-fitting frequency bands of power spectrum density of surface electromyography for type I and type II fibers in rats’ leg muscles by changing the frequency band boundaries. Secondly, we conducted tests on the human body and collected the electromyography of the atrophied muscles of the subjects over a period of 21 days. The changes in muscle fiber composition were evaluated using the frequency bands of power spectrum density obtained from rat experiments. The method was to evaluate the changes in type I fibers by the changes in the area of the best-fitting frequency band of type I fibers and to evaluate the changes in type II fibers by the changes in the area of the best-fitting frequency band of type II fibers. Results: The results of rat experiments showed that type I fibers best fit the frequency band of 20–330 Hz and type II fibers best fit the frequency band of 176–500 Hz. The results of human testing showed that the atrophy of the two types of fibers was consistent with the changes in the areas of the corresponding best-fitting frequency bands. Conclusions: The test results demonstrate the feasibility of using surface electromyography to evaluate muscle fiber-type composition and subsequently assess muscle atrophy. Further research may contribute to the diagnosis and treatment of disuse muscle atrophy.

## 1. Introduction

Disuse muscle atrophy is a skeletal muscle dysfunction usually caused by reduced muscle contraction activities and reduced muscle loads. The common cause of disuse muscle atrophy is limb immobilization caused by plaster coating after fracture surgery, which leads to muscle atrophy while the fracture heals. Muscle atrophy can affect the function of motor units and limit habitual activities in daily life [1,2,3,4]. The severity of disuse muscle atrophy increases with increasing immobilization time and skeletal muscles exhibit some biochemical and histological changes, including decreased muscle mass and cross-sectional area (CSA) [5,6], abnormalities in enzymes and mitochondria, and changes in muscle fibers [7,8]. These changes can lead to a gradual decrease in muscle strength and durability [8,9], which is most pronounced in lower limb muscles.

Disuse muscle atrophy affects the normal life of patients, and timely treatment can effectively slow down the process of pathological changes and promote the recovery of muscle atrophy [10,11,12,13]. Accurately and objectively assessing muscle atrophy is of great help for clinical treatment and rehabilitation. However, clinical evaluation typically depends on qualitative grade indicators such as limb circumference, local soft tissue tension, and muscle contraction strength and persistence. There is usually subjectivity in the doctor’s judgment, which cannot achieve objective and accurate quantitative evaluation [14,15,16,17]. There are also some invasive measurement methods, such as needle electromyography, which, although quantitative and more accurate, can cause harm and pain to patients [18,19]. Unlike invasive measurement methods, there are also some commonly used non-invasive methods, such as MRI (Magnetic Resonance Imaging) [20] and ultrasound [21], which can be used to examine the muscle condition of patients. Surface electromyography (sEMG), as a non-invasive method and the main research object of this paper, has been widely used in the detection and treatment of muscle and nervous system diseases in recent years [22]. MRI and ultrasound mainly reflect the morphology of muscle tissue structure through images. sEMG examines the bioelectric activity on the surface of muscle skin. MRI, ultrasound, and sEMG have their own focuses and often form complementary technical means to help doctors examine the patient’s muscle condition.

Skeletal muscle fibers can be classified into slow muscle fibers (type I fibers) and fast muscle fibers (type II fibers) based on their contraction rate. The characteristics of fibers are determined by the main isoforms of myosin heavy chain (MHC), and type II fibers include subtypes IIa, IId/x, and IIb [23,24]. As muscle atrophy progresses, the muscle volume, mass, and CSA of fibers decrease. After 48 h of hind-limb suspension, the muscle mass decreases by 20% and the CSA of the soleus muscle decreases by 9% in mice [5]. After 7 days of bed rest, the thigh muscle volume of the subjects decreases by 3% [25]. The suspension of hind limbs for 7 days results in the CSA of type I and type IIb fibers in the soleus muscle being decreased by 33% and 16%, respectively [26]. After 11 days of space flight, the decrease in the CSA of fibers in the lateral thigh muscle is more pronounced in type IIb, followed by type IIa, and the least significant is type I fibers [27]. The fiber-type composition of atrophic muscles has also changed. After 14 days of hind-limb suspension in rats, the MHC I subtype in the soleus muscle significantly decreases, while the MHC IIa or IIx subtype increases [7]. After 4 weeks of limb immobilization, the muscles mainly composed of type I fibers have the characteristics of type II fibers in the human skeletal muscles [28]. Reduced skeletal muscle mass, reduced CSA of muscle fibers, transition from slow to fast muscle fibers, and changes in functional characteristics were observed in different types of muscles under disuse conditions [5,7,25,26,27,28,29,30,31,32,33]. Therefore, the condition of disuse muscle atrophy can be evaluated by evaluating the fiber-type composition of the atrophic muscle, based on the changes in type I and type II fibers during muscle atrophy.

Some studies support the possibility of using surface electromyography as an effective tool for evaluating the fiber-type composition of atrophic muscle. In fact, functional relationships between fiber-type composition and response in the spectrum of the sEMG have been documented by comparing frequency domain parameters of sEMG between rats composed of different fiber types [34] or analyzing changes in the activity of muscles with different fiber composition [35]. There is a significant proportion of type II fibers in the skeletal muscles of patients with chronic obstructive pulmonary disease (COPD) [36,37,38,39]. Some studies [40,41,42,43] found that compared with healthy individuals, the sEMG power spectrum of skeletal muscles in patients with COPD shifts towards higher frequencies. In these studies on COPD, the sEMG power spectrum of muscles with higher type II fiber content shifted towards higher frequencies, while the power spectrum distribution at lower frequencies was mainly related to muscles with higher type I fiber content. However, due to the use of fatigue motion tasks in these studies on COPD, the degree of damage to the muscle function and physical performance of patients may worsen during fatigue tasks, thereby limiting the ability of surface electromyography analysis to reliably detect changes in muscle fiber composition [41,44,45,46]. Considering the disadvantages of fatigue motion tasks, the study of A. Casabona [47] adopted non-fatiguing motion tasks, and experimental results showed that a higher proportion of type II fibers corresponded to higher spectrum parameters (median frequency and mean power frequency), which means the power spectrum shifted towards high frequencies. In summary, the quantitative relationship between fiber-type composition and sEMG can be further explored.

Change in fiber-type composition is the key feature of disuse muscle atrophy, so the key point of evaluating disuse muscle atrophy lies in the evaluation of fiber-type composition. The necessity of using an objective and non-invasive sEMG method to evaluate disuse muscle atrophy is reflected in the fact that sEMG can be combined with rehabilitation training and electrical stimulation to form a treatment strategy of sEMG feedback. By using a quantitative and non-invasive sEMG method to evaluate fiber-type composition, on the one hand, it can guide treatment strategies, and on the other hand, it can evaluate the therapeutic effects of treatment strategies. Specifically, targeted treatment strategies can be formed, and the treatment strategies for disuse muscle atrophy can be optimized to achieve better treatment effects. The goal of this paper is to find the corresponding relationship between type I fibers, type II fibers, and sEMG by analyzing the actual fiber-type composition and sEMG of rats. Therefore, the fiber-type composition can be non-invasively evaluated when sEMG is collected, and then disuse muscle atrophy can be evaluated. Furthermore, we can apply the correspondence between the fibers and sEMG of rats’ muscle to human muscle, and analyze the atrophy degree of human muscle fibers through the sEMG of human atrophic muscles.

In this study, in order to explore the quantitative relationship between the fiber-type composition and power spectrum density (PSD) of sEMG, we collected electromyography and obtained the actual fiber-type composition of rats’ muscles through muscle biopsy and fluorescence staining. We determined two PSD frequency bands of sEMG corresponding to type I fibers and type II fibers by changing the frequency band boundaries. In order to verify the correspondence between the PSD frequency bands and muscle fibers, surface electromyography of atrophic muscles of humans was collected under non-fatigue tasks (considering the shortcomings of fatigue tasks). During the 21-day experimental period, the changes in the area of two PSD frequency bands of human atrophic muscles’ electromyography were recorded and the changes in the frequency band area were used to evaluate the changes in the corresponding fiber composition.

## 2. Materials and Methods

### 2.1. Animal Experiments

#### 2.1.1. Animals and Ethics

Twenty-five male Sprague-Dawley rats aged 8–10 weeks and weighing 200–250 g were obtained from Hangzhou Medical College. All rats were placed in a room in which the temperature and light/dark were cyclically controlled for one week before the experiment. All animal handling procedures and experimental protocols were approved by the Ethics Committee of Laboratory Animal Welfare of the Laboratory Animal Center of Zhejiang Province (Approval No. ZJCLA-IACUC-20010455).

#### 2.1.2. Disuse Atrophy Modeling

**Figure 1:**
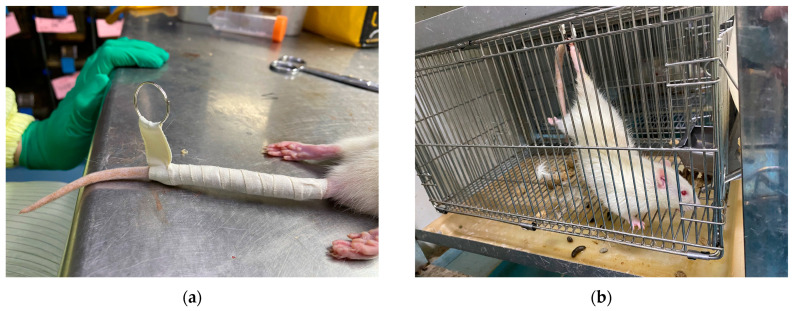
Methods to achieve hind-limb atrophy in rats. (**a**) Fix a metal keyring to the tail of the rat. (**b**) Suspension of the rat’s hind limbs through the metal keyring at the tail.

As shown in Figure 1, the disuse atrophy modeling used the method of suspending the tail of the rat, which achieved hind-limb atrophy through hind-limb suspension [48]. Before suspension, the rats were weighed and anesthetized. Figure 1a shows fixing a metal keyring to the tail of the rat. Figure 1b shows hind-limb suspension of the rat using the metal keyring on the tail of the rat. The rats could carry weight and move through their forelimbs, but their hind limbs could not touch the ground and cage walls. All rats were kept in a quiet and temperature-controlled environment.

#### 2.1.3. Experimental Design

**Figure 2:**
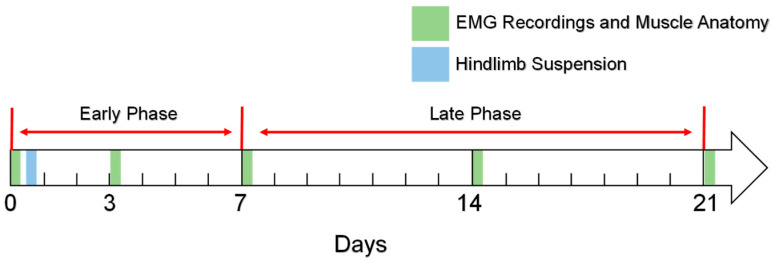
Timing of experimental events.

As shown in Figure 2, 25 rats were randomly divided into five groups with five rats in each group. The suspension time for each group of rats was 0 days, 3 days, 7 days, 14 days, and 21 days, respectively. After the suspension of the hind limbs of each group of rats, anesthesia was administered to the rats. Afterwards, electromyography (EMG) recordings and muscle anatomy (muscle anatomy is used to measure fiber-type composition) were performed on the rats.

#### 2.1.4. EMG Acquisition and Processing

The collection of electromyography was accomplished through sensor equipment designed and manufactured by our group, and the core parts were mainly flexible electrodes and collection circuits. In addition, we have developed a matching software, which transmits data with the sensor equipment through the serial port. The main purpose of this software is to visualize the sEMG data collected by the sensor equipment, and so we could see the waveform and value of the collected sEMG signal in real time.

Details of principles and procedures of the fabrication process for the flexible electrode are mentioned in our previous paper [49]. Briefly, the flexible electrode is covered by Polydimethylsiloxane (PDMS), with its conductive circuit part printed on the flexible polyimide (PI) substrate. The designed electrode part is gilded with gold to improve conductivity and the size is set as a 0.9 cm × 0.4 cm rectangle, which we adjusted to 4 cm × 4 cm in the later applications on patients. The whole flexible electrode has excellent biocompatibility, great chemical stability, flexibility, and stretchability, which is quite crucial to adjust to the variability of the complex circumstances. Tests were carried out to test the stretchability and reliability of this flexible electrode. The stretchability test was carried out by applying a uniaxial strain to both sides of the flexible electrode to explore the maximum length without breaking. After testing, the flexible electrode had a stretching capacity of more than 30%, which exceeds the normal strain of human skin (30%), and can fit comfortably and tightly to the skin. The mechanical reliability test was performed by twisting the flexible electrode at an angle of 30° hundreds of times. After each bend, the whole system’s SNR (signal/noise ratio) was evaluated by EMG signal acquisition.

The acquisition chip used in the acquisition circuits was the ADS1299 chip produced by Texas Instruments (TI). This acquisition chip has low noise characteristics, a 24-bit ADC (Analog-to-Digital Converter), a sampling rate of up to 16k SPS, and a common mode rejection ratio (CMRR) of up to −110 dB.

We used a professional EMG signal simulator to test the acquisition performance of the entire system. The test indicators and test methods refer to the pharmaceutical industry standard YY/T 1095-2015 of the People’s Republic of China [50]. In terms of amplitude accuracy, the measurement range can reach ±5 mV, and the error is no more than ±10% or ±2 μV (the maximum value of the two). The system resolution (test sensitivity) is ≤2 μV. The root mean square value (RMS) of the system noise is ≤1 μV. The passband of the system is not narrower than 20–500 Hz (based on the −3 dB point).

Because rats cannot perform actions according to human settings, the conventional method of collecting rats’ sEMG is to collect M waves through electrical stimulation. In order to obtain the electromyography of rat’s leg muscles, stimulation of the sciatic nerve was used to induce leg electromyography [51].

**Figure 3:**
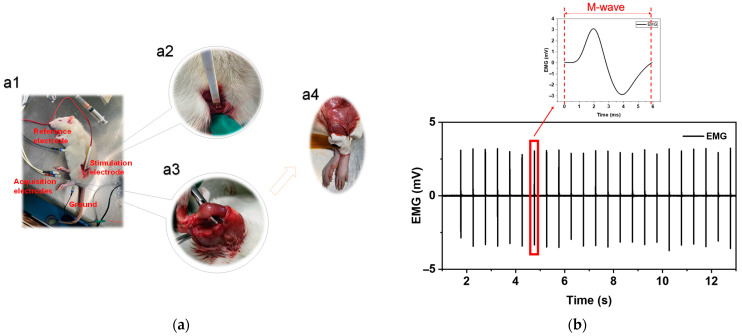
(**a**) (**a1**) The positions of electrodes during electromyography acquisition on rats. (**a2**) The hook-shaped stimulation electrode. (**a3**) The muscle of the rats on which we performed the EMG acquisition. (**a4**) The flexible surface electrode. (**b**) The representative traces of an electrical stimulation-induced EMG wave.

As Figure 3a shows, after blunt dissection of the muscles and soft tissues around the femur of anesthetized rats, the sciatic nerve that controls lower limb muscle movement and contraction was exposed. The hook-shaped stimulation electrode was placed on the sciatic nerve under the femur and positioned 10 mm proximally to its trifurcation. The use of hook-shaped stimulation electrodes ensures contact with nerves and provides good protection. After removing the fur of the rat’s legs using a shaver and fur removal cream specifically for rats, they were disinfected and cleaned with a 75% ethanol solution. Finally, the flexible electrodes as the collection electrodes were placed at the muscle belly to collect data, respectively, on the upper and lower 0.5 cm from the midpoint of the muscle. The electrical stimulation signal was generated by the electrical stimulation device named Plexon from the Neuroscience Technology company of the USA. Bipolar square waves (amplitude: 2 mA, frequency: 2 Hz, pulse width: 100 µs, duration time: 30 s) to rouse slight movement of the lower limbs of the rats are delivered by the stimulation electrodes, and the triggered responses of the extensor digitorum longus (EDL) and soleus (SOL) muscle are acquired by the collection electrodes. The use of the bipolar square waves is to reduce stimulus artifacts. After each electrical stimulation, the available M wave of EMG will closely follow the stimulus artifact. Figure 3b shows M waves evoked by electrical stimulation.

The selection of the electrical stimulation amplitude and frequency is to induce obvious but not violent movement of the rat’s muscles through low-frequency stimulation pulses. High-frequency stimulation pulses may be too frequent. The electrical stimulation amplitude can be gradually increased from a small value until the desired movement of the rat’s muscles is seen. After the stimulation frequency is determined, the appropriate pulse width is set according to the value of the stimulation frequency. The duration time of the electrical stimulation is considered to ensure that sufficient sEMG data are collected.

The evoked sEMG of the SOL and EDL was collected (sampling rate: 16 kHz). The raw data were passed through a 50 Hz notch filter to remove power frequency noise. Typically, the frequency contents of sEMG range between 6 and 500 Hz, and the power spectrum is mainly concentrated between 20 and 150 Hz [52]. Since there may be a lot of low-frequency noise below 20 Hz, data after removing power frequency noise were passed through a 20–500 Hz bandpass filter to adjust the signal to the effective EMG frequency range. Finally, the PSD and median frequency (MF) of the effective EMG signal were calculated, and the calculation of MF is shown in Equation (1).

(1) $$\sum_{j=20Hz}^{MF}P_{j}=\sum_{j=MF}^{500Hz}P_{j}=\frac{1}{2}\sum_{j=20Hz}^{500Hz}P_{j}$$

where $P_{j}$ is the power spectrum of EMG signal.

#### 2.1.5. Fiber-Type Composition

**Figure 4:**
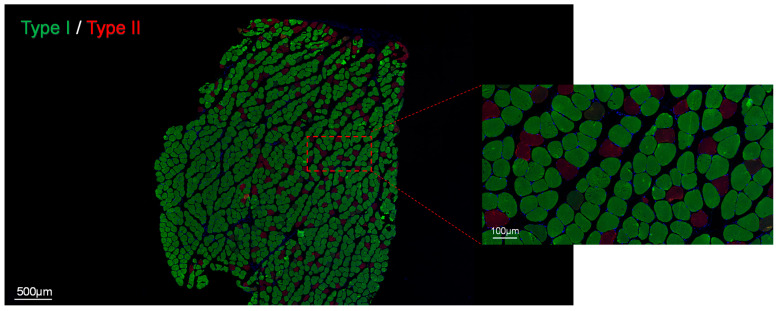
Immunofluorescence staining of fibers.

After the electromyography measurement was completed, the rats were killed by cervical dislocation and the SOL and EDL were immediately dissected. We used immunofluorescence staining to evaluate the fiber-type composition of the muscles [53]. Firstly, immunofluorescence staining of type I and type II fibers was performed. Immunofluorescence staining of type I (myosin heavy chain 7, MYH7) and type II (myosin heavy chain 1, MYH1) muscle fibers was performed according to the following protocol. Briefly, after antigen recovery, circulation, and 3% BSA blockade, the muscle tissue slices of the sample were incubated overnight with the first antibody at a temperature of 4 °C. After washing with PBS, the sample was incubated with the second antibody at room temperature and in the dark for 40 to 50 min. Finally, DAPI solution was used to label the cell nucleus. We diluted rabbit anti-MYH1 polyclonal antibody at a ratio of 1:200 to detect MYH1. We diluted rabbit anti-MYH7 polyclonal antibody at a ratio of 1:200 to detect MYH7. Omitting primary antibodies was used to prepare negative staining control. As shown in Figure 4, type I fibers and type II fibers were dyed green and red, respectively, after dyeing. Next, we used ImageJ software to analyze the density and average fiber area of muscle fibers. The density of fibers was analyzed using the counting tool of ImageJ software. After replacing the dyeing results with an 8-bit bitmap, the area of the red and green areas was distinguished through the threshold analysis function.

After obtaining the fiber-type composition of rats by fluorescent staining, the mean fiber area was calculated by Equation (2).

(2) Mean Fiber Area=S∕N

where *S* is the total area of a given fiber in this muscle and *N* is the total number of a given fiber in this muscle.

We set the health level of type I and type II of the five rats on day 0 as 1.0, and the health level of the two fibers of rats with other suspension times was calculated by Equations (3) and (4) (the muscle studied was only the SOL muscle).

(3) Health Level−Type I=Area_TypeI ∕ MeanArea_TypeI_day0

(4) Health Level−Type II=Area_TypeII ∕ MeanArea_TypeII_day0

where MeanArea_TypeI_day0 is the mean area of type I fibers in 5 rats on day 0 and MeanArea_TypeII_day0 is the mean area of type II fibers in 5 rats on day 0. Area_TypeI is the area of type I fibers and Area_TypeII is the area of type II fibers in a given rat.

### 2.2. Human Testing

#### 2.2.1. Subject Selection

For patients with bone and joint injuries, the most severe period of disuse muscle atrophy is between the completion of surgery and one month after the brace is fixed. However, due to differences in the nature of patient follow-up and irregular reexamination times, it is difficult to ensure that data at corresponding time points are collected and the data interval may be relatively large. Therefore, the subjects for this experiment on disuse atrophy were healthy people who had strengthened their biceps muscles through dumbbell exercises and stopped exercising during the experiment. The duration of the experiment was specified as 21 days and there were 12 test subjects (gender: 4 women and 8 men, age: 26.0 ± 3.0 years).

#### 2.2.2. EMG Acquisition and Processing

The system used for human sEMG acquisition was consistent with that described in the rat experiments.

sEMG testing was performed in a quiet environment. Differential electrodes were placed on the biceps brachii muscle. After cleaning the surface of the biceps brachii with 75% ethanol, differential electrodes were placed above and below the muscle belly 2 cm from the midpoint of the muscle, with the reference electrode placed on the bone at the wrist. During signal acquisition, subjects were asked to perform 10 upper arm curl movements at maximum free contraction force.

Surface electromyography of the human body was passed through a 50 Hz notch filter to remove power frequency noise and the effective spectrum range of EMG was obtained through a band-pass filter of 20–500 Hz (sampling rate: 16 kHz). Next, the PSD of surface electromyography was calculated.

## 3. Results

### 3.1. Animal Experiments

#### 3.1.1. Fiber-Type Composition

**Figure 5:**
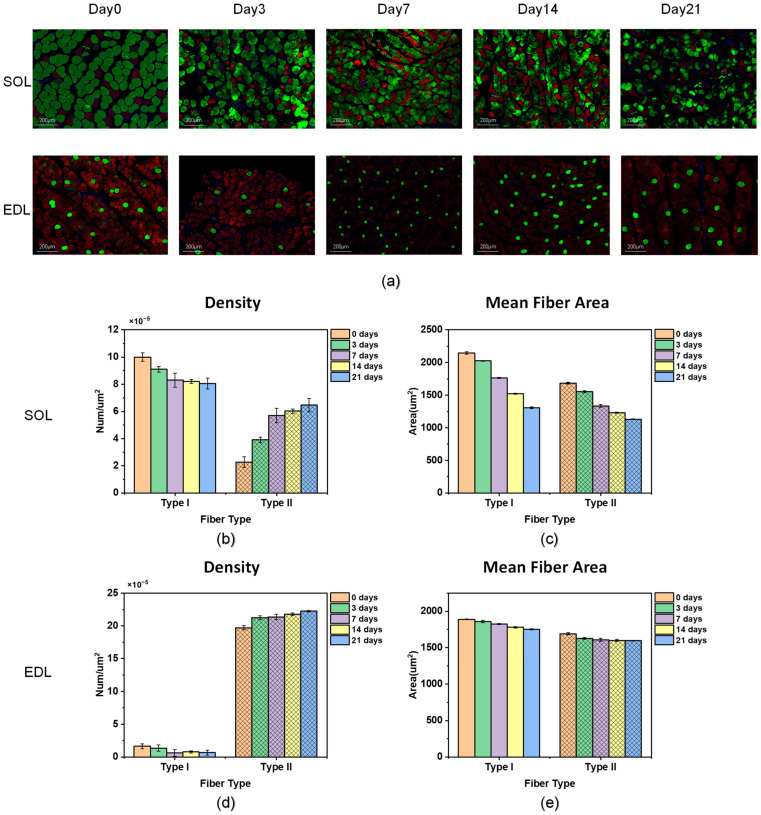
Fiber-type composition of the SOL and EDL. (**a**) Representative pictures of staining of the SOL and EDL at five time points (type I fiber: green, type II fiber: red). (**b**) Density changes of two fibers of the SOL at five time points. (**c**) Mean fiber area changes of two fibers of the SOL at five time points. (**d**) Density changes of two fibers of the EDL at five time points. (**e**) Mean fiber area changes of two fibers of the EDL at five time points.

Fluorescent staining was used to evaluate the fiber-type composition of rats’ muscles, and the results are shown in Figure 5. Figure 5a is a representative figure of the staining of each suspension group under the microscope. It can be seen from the figure that the EDL is mainly composed of type II fibers (red), while type I fibers (green) account for a larger proportion in the SOL. There are significant differences in the fiber-type composition of the SOL and EDL, so we selected these two muscles for experiments.

There is one suspension group for each of the five suspension times and there are five rats in each suspension group. It should be noted that each bar and its corresponding error bar in Figure 5b–e represent the mean and standard deviation of five rats’ values. As shown in Figure 5b,d, type II fiber density increases and type I fiber density decreases with increasing suspension time. This suggests a shift from type I fibers to type II fibers as muscle atrophy progresses and this shift is a distinguishing mark of disuse muscle atrophy. The changing trend of the fiber density of type I fibers and type II fibers in the EDL is not as obvious as that in the SOL, probably because the proportion of type I fibers in the EDL is smaller. In other words, since there is a shift from type I fibers to type II fibers during muscle atrophy and the SOL has a larger proportion of type I fibers, it can be seen that the density of type I fibers shows a greater decreasing trend and the density of type II fibers shows a greater increasing trend in the SOL. Figure 5c,e shows the mean fiber area changes of the two fibers of the EDL and SOL at five time points. The mean fiber area of both type I fibers and type II fibers decreases with increasing suspension time, both in the SOL and the EDL, which is consistent with the development of muscle atrophy. Since type I fibers contract slowly but are not prone to fatigue, type I fibers mainly affect endurance. Since type II fibers contract quickly but fatigue easily, type II fibers mainly affect explosive power [54]. The results show that as muscles atrophy, type I fibers atrophy more severely (there is a shift from type I fibers to type II fibers), which suggests that muscle atrophy affects endurance more than explosive power.

#### 3.1.2. Analysis of sEMG

**Figure 6:**
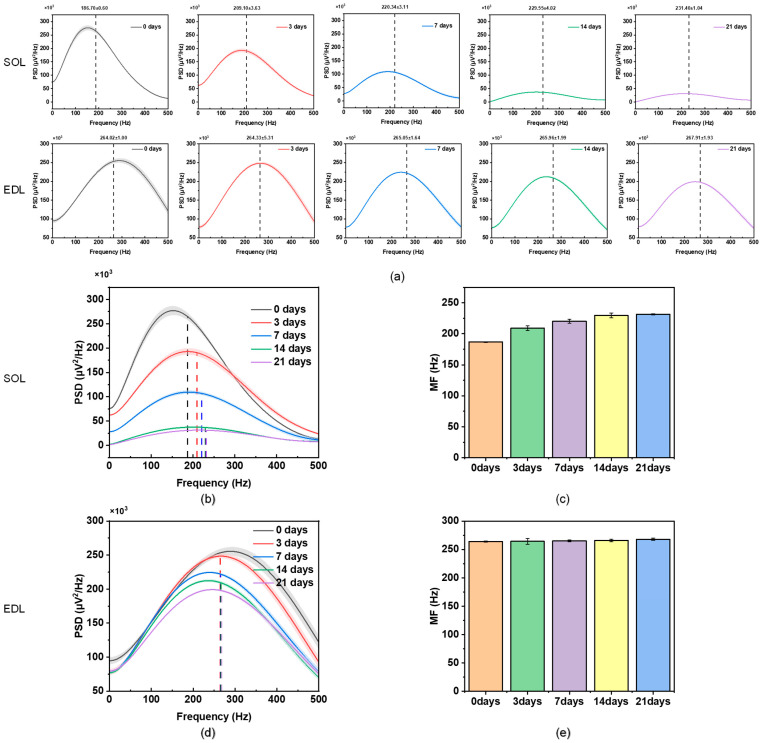
(**a**) PSD of EMG and MF (dashed line) of the SOL and EDL at five time points. (**b**) PSD of the SOL at five time points (a merging of the first row in (**a**), the dashed line identifies the MF of each curve). (**c**) MF of the SOL at five time points. (**d**) PSD of the EDL at five time points (a merging of the second row in (**a**), the dashed line identifies the MF of each curve). (**e**) MF of the EDL at five time points.

Figure 6 shows the mean PSD and MF of the SOL and EDL under different suspension times (since there are five rats in the suspension group at each time point, the error bands represented by the shaded areas and error bars in the figure should be noted). According to the PSD distribution and corresponding MF, as the suspension time increases, the degree of atrophy gradually increases and the PSD of the SOL muscle obviously shifts to the high-frequency band, while the PSD of the EDL muscle basically does not shift.

**Figure 7:**
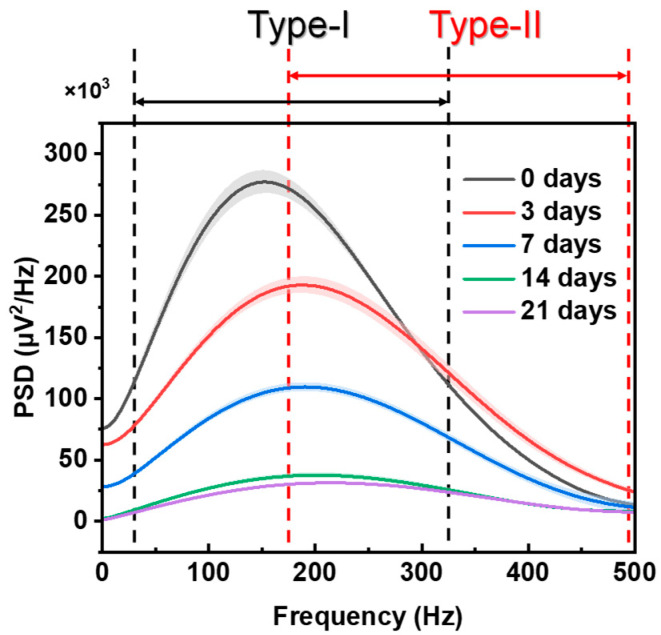
Different contributions of type I and II fibers to the PSD.

As shown in Table 1, we compared the experimental results of the SOL and EDL. These resultant observations stimulate the hypotheses that different muscles have a specific PSD of sEMG and type I and type II fibers contribute differently to the PSD. Therefore, we proposed the inference of frequency demarcation to quantitatively analyze the different contributions of muscle fibers to the high-frequency and low-frequency components of the PSD. As shown in Figure 7, the lower frequency band was mainly influenced by type I fibers and the higher frequency band was mainly influenced by type II fibers. Therefore, in Figure 6b,c, the apparent shift of the PSD of the SOL to the higher frequency band was due to the shift from type I fibers to type II fibers during muscle atrophy (the SOL has a higher content of type I fiber). In Figure 6d,e, although type I fibers also shift to type II fibers during EDL muscle atrophy, there is no obvious shift in the PSD of the EDL because type II fibers are the main component in the EDL.

**Figure 8:**
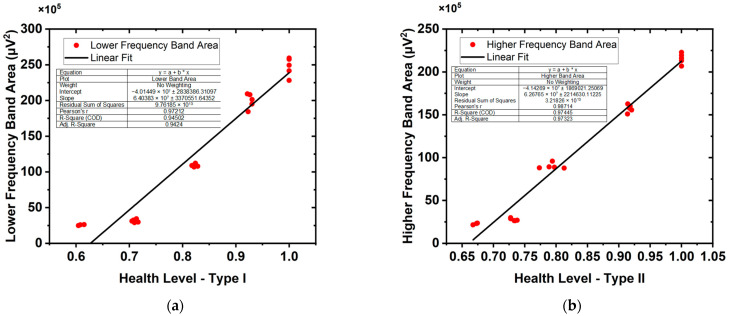
The best fit of PSD frequency band area and the health level of the fiber area in the SOL. (**a**) Fitting of the lower frequency band (20–330 Hz) area of the PSD and the health level of the fiber area of type I fibers. (**b**) Fitting of the higher frequency (176–500 Hz) band area of the PSD and the health level of the fiber area of type II fibers.

As shown in Figure 8, based on the assumption that muscle fibers contribute differently to the high- and low-frequency components of the PSD, the frequency scanning method (continuously changing the frequency band boundaries) was used to calculate the fitting coefficient between the respective areas of the higher and lower frequency bands and the health level of the fiber area, and determine the best fitting boundaries of the higher and lower frequency bands. The muscle studied in Figure 8 is only the SOL muscle. When the PSD frequency band is set to 20–330 Hz, the area of the PSD frequency band best fits the health level of the fiber area of type I fibers, while if the PSD frequency band is set to 176–500 Hz, the area of the PSD frequency band best fits the health level of the fiber area of type II fibers. Experiments on the legs of different rats show some fluctuations in the band boundary, but the fluctuations are minor. The high correlation indicates that we can use frequency band areas of PSD to predict the health level of different fiber types.

### 3.2. Human Testing

**Figure 9:**
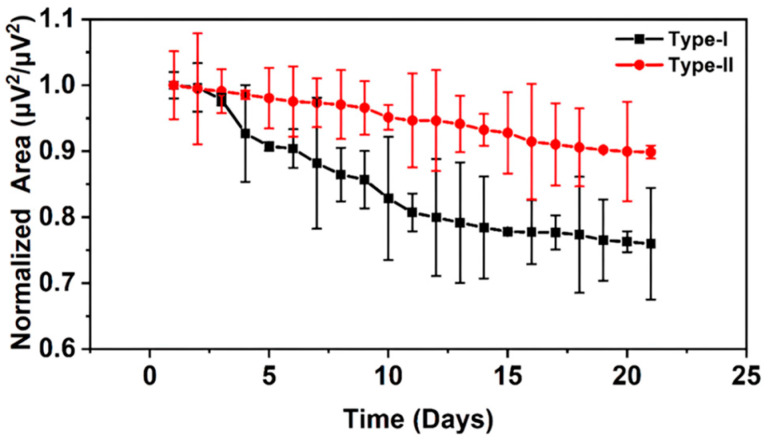
Changes in the normalized area of the PSD frequency bands corresponding to type I fibers and type II fibers with time.

In animal experiments, we obtained the boundaries of two PSD frequency bands corresponding to type I fibers and type II fibers, respectively. Next, sEMG of the human body was analyzed based on these two PSD frequency bands. As shown in Figure 9, the normalized areas of the two PSD frequency bands corresponding to type I fibers and type II fibers were calculated. Among them, the value of the first day was used as the standard for normalization. Values for each day represent the average across subjects and error bars show the standard deviation. It can be seen from the figure that the normalized frequency band areas of the PSD corresponding to type I fibers and type II fibers both decrease with time, but the normalized frequency band area of the PSD corresponding to type I fibers decreases more. Muscle biopsies can be taken from humans to determine fiber-type composition and thus determine the atrophy degree of muscle fibers over time. The actual atrophy degree of the fibers is compared with the PSD frequency band characterization method to determine the feasibility of the PSD frequency band characterization method. However, muscle biopsy can cause harm to the human body and should not be adopted. It is known from animal experiments that during the process of muscle atrophy, both type I fibers and type II fibers atrophy, and type I fibers atrophy more severely due to the transformation to type II fibers. The results in Figure 9 are basically consistent with the changes in fiber-type composition in rats during atrophy.

## 4. Conclusions and Discussion

In this work, we used sEMG to achieve quantitative assessment of disuse muscle atrophy.

Firstly, a rat model of disuse muscle atrophy was established by hind-limb suspension. For rats of each suspension time, sEMG induced by bipolar square waves was collected and the fiber-type composition of the muscle was obtained by fluorescent staining. According to the changes in fiber density and the mean fiber area of the SOL and EDL over time, it can be concluded that during the process of muscle atrophy, both type I and type II fibers atrophied and type I fibers transformed into type II fibers. According to the analysis of the PSD of rats’ sEMG, it was observed that with the increase in the degree of atrophy, the PSD of the SOL shifted significantly to higher frequencies, while the PSD of the EDL basically did not shift. According to the difference in fiber-type composition between the SOL and EDL, it is speculated that it may be because the content of type II fibers in the EDL is higher and the change in type I fibers of the lower proportion has little effect on the PSD. According to the different contributions of the two fibers to the PSD, we divided two PSD frequency bands corresponding to type I fibers and type II fibers and fitted the optimal band boundaries through results of EMG and fiber-type composition.

Afterwards, sEMG of human muscles was collected and the frequency band boundaries obtained from the rat experiments were applied to human sEMG. According to the results of human testing, it can be seen that the changes in the frequency band area of PSD and the fiber-type composition are consistent.

It is obviously worth noting why the frequency band boundaries obtained in rat experiments can be transferred to humans. The frequency band boundaries of human sEMG may be different from rats’ sEMG. In future studies, researchers will be able to improve the method presented in this paper by increasing the sample size and refining the fitting model. However, the method of associating slow and fast muscles with frequency bands of sEMG has innovation and practical value. This work can serve as a basis for sEMG to assess the state of muscle atrophy and further research may help in the diagnosis and treatment of disuse muscle atrophy.

The small sample size is a limitation of this study. Although we attempted to select rats of similar weight and size, the differences in fiber-type composition within the same group may still limit the study. Future studies could consider expanding the sample size and considering how to address the impact of differences between rats. At the same time, human variability factors like age, gender, and muscle condition, which may affect clinical applicability, should be further considered. Future studies should also explore these variables and refine frequency boundaries to better align with human muscle properties, addressing limitations in directly extrapolating from rat models.

The differences across species have caused limitations, but the idea of assessing the fiber-type composition of muscles through sEMG can be further explored in future studies. In this paper, we fitted two frequency bands corresponding to type I and type II fibers in the PSD of sEMG, and the complexity of the entire fitting model was not high. In the next step, we intend to further study the characteristics of muscles and the physiological basis of sEMG and establish a more complex and better fitting model to obtain a better correspondence between sEMG and fiber-type composition. At the same time, the assessment of the fiber-type composition of the entire muscle through single-channel sEMG has obvious limitations, because the fiber-type composition of the muscle and sEMG are different at different locations in the muscle. Future studies can consider incorporating high-density surface electromyography [55,56], which can provide a more refined assessment of the entire muscle. High-density surface electromyography analyzes muscles in planar space and time through the sEMG matrix, which can obtain more accurate analysis results for muscle fiber-type composition.

The method based on sEMG in this paper can be used as a supplement to existing methods. As mentioned earlier, MRI and ultrasound focus on imaging the muscles, while sEMG focuses on examining the electrical activity of the muscles. The combination of these technical means can obtain more accurate muscle examination results. The method in this paper also has some unique advantages. First of all, MRI and ultrasound require the help of large and expensive examination equipment in hospitals and may also require the support of professionals. However, with the development of sensor technology, many sEMG detection devices are small in size and affordable for ordinary people. In recent years, sEMG detection equipment has gradually become a trend for home use, such as use in the field of fitness (users observe the degree of muscle exertion through changes in sEMG). Secondly, the contents of this paper can be further studied in the future to form a system that combines treatment and evaluation. The patients’ atrophied muscles can be treated through feedback training and feedback electrical stimulation based on sEMG, and then the effect of the treatment can be evaluated through the relationship between fiber-type composition and sEMG. This system that combines treatment and evaluation is developed by professionals, and with the product manual, it is possible for patients to use it at home.

## Figures and Tables

**Table 1:** Comparison table of the experimental results of the SOL and EDL.

Term/Muscle		SOL	EDL
Main fiber component		Type I	Type II
Fiber changes of atrophy process	Common	Mean fiber area of both type I and type II decreases Type II density increases and type I density decreases A shift from type I to type II	
	Difference	Changing trend of fiber density in the SOL is greater	
PSD changes of atrophy process		Obviously shifting to the high frequency	Basically no shift

## Data Availability

The data presented in this study are available on request from the corresponding author.
